# Ethnopharmacological Inspections of Organic Extract of* Oroxylum indicum* in Rat Models: A Promising Natural Gift

**DOI:** 10.1155/2019/1562038

**Published:** 2019-04-03

**Authors:** Mst. Marium Begum, Azharul Islam, Rayhana Begum, Md. Sahab Uddin, Md. Sohanur Rahman, Sumiya Alam, Wahida Akter, Munny Das, Md. Sohanur Rahman, A. H. M. Rahmatullah Imon

**Affiliations:** ^1^Department of Pharmacy, East West University, Dhaka, Bangladesh; ^2^Department of Pharmacy, Primeasia University, Dhaka, Bangladesh; ^3^Department of Pharmacy, Dhaka International University, Dhaka, Bangladesh; ^4^Department of Pharmacy, Southeast University, Dhaka, Bangladesh; ^5^Graduate School of Innovative Life Science, University of Toyama, Toyama, Japan; ^6^Department of Pharmacy, Atish Dipankar University of Science and Technology, Dhaka, Bangladesh; ^7^Department of Mathematical Sciences, Ball State University, Muncie, USA

## Abstract

The stem bark of* Oroxylum indicum* (*O. indicum*) was aimed at testing for anti-inflammatory, antiulcerative, antihyperglycemic, and antidyslipidemic activities. Liver enzyme concentration (SGPT, SGOT) had also been assessed. After being extracted in organic solvent, 3 distinct doses, 100, 200, and 400 mg/kg b.w. (p.o.), were used. For edema formation 0.1 ml carrageenan at a dose of 1% w/v was injected into paw of left hind. It showed a fall of edemas 37.50%, 48.34%, and 55.83% while used doses were 100, 200, and 400 mg/kg b.w. (p.o.) individually. The EtOH extract of* O. indicum* (50%) and its fractions PET, CLF, EtOAc, and nBUT were studied against ethanol-induced gastric mucosal damage. Only PET and n-BuOH exhibited the highest percentage of protection and were 96% and 99%, respectively, persuaded by ethanol. In OGTT glibenclamide revealed reduction of glucose level to 7.55 ± 0.22 mmol/L from 10.57 ± 0.32 mmol/L after 30 minutes. Antihyperglycemic activities were assessed for 8- and 12-week duration in diabetic rats. Glibenclamide reduced glucose level from 33.50±0.31 to 7.90±0.19 mmol/L in 12 weeks. In 12 and 8 weeks, combination therapy lowered blood glucose level to a normal extent by 79% and 61% individually. In antidyslipidemic activities after 12-week treatment, it revealed simvastatin; MEOI (400 mg/kg b.w.) and combination of both reduced TC level by 44%, 28%, and 48% consequently followed by TG and LDL. In 8-week treatment, HDL levels were increased by 34%, 13%, and 36%, and in 12 weeks increased by 36%, 8%, and 38% consequently. Liver enzyme concentration after 12 weeks of treatment with glibenclamide, 400 mg/kg b.w. (p.o.) of MEOI and combination of both, exhibited the fact that concentration of SGPT showed downturn by 43.23%, 8.01%, and 54.86% and SGOT by 42.40%, 5.31%, and 44.85%. This study remarked that* O. indicum* has anti-inflammatory, antiulcer, antidiabetic, and antidyslipidemic potentials but has no ameliorative effect on liver enzyme.

## 1. Introduction

The trumpet tree* Oroxylum indicum* (*O. indicum*) is found in the tropical region of South East Asian counties like India, Japan, China, Sri Lanka, Malaysia, and Bangladesh [1]. This magnificent plant has many names like Broken Bones, Midnight Horror, Sonapatha, Dashmula, and Shyonaka. This tree is immensely valued in India for its various ayurvedic preparations [2]. It is used as key ingredients of mostly used ayurvedic preparation “Dasamula;” it means ten roots. The whole plant is used as a utilizable part for medicinal value [3]. In folk medicine practices* O. indicum* has the significance as being an astringent, carminative, blood purifier, tonic diuretic, and laxative, as well as other problems like stomach complaints such as diarrhea, dysentery, and allergic dermatitis [4]. The plant parts, especially seed, ripen fruits, both stem and root bark, and leaves, are cherished for various ayurvedic formulations such as Amritarishta, Dantyadyarista, Narayana Taila, Dhanvantara Ghrita, Brahma Rasayana, and Chyawanprash Awaleha [5]. Those ayurvedic preparations are used for inflammation, ulcer, cancer, diarrhea, fever, jaundice, arthritis, and oxidative stress. But all ethnomedical uses of this plant are not experimentally proved by modern technology and research experiments.

In ayurveda and folk medicine this plant part is used as medicinal agents for various disorders such as cancer, diarrhea, diabetes, fever, bone pain, ulcer, and jaundice but this entire claim is not scientifically proved [6–8]. Fruits pods were extensively reported for inhibition of adipogenesis and lipase activity [9]. Seed contained many flavonoids including baicalein-7-*O*-gentiobioside and other baicalein derivatives which include 7-*O*-glucoside, 7-*O*-glucuronide, 5,6,7-trimethoxyflavone-8-O-*β*-D-glucopyranoside, oroxylin A-7-O-*β*-D-glucuronide butyl ester, 6-methoxy-baicalein, oroxylin-A-7-O-glucoside, 5,7-dihydroxy–flavone, uracil, baicalein 6-methoxy-7-glucuronide, stilbenoids (E)-dihydropinosylvin-2-carboxyl-5-O-*β*-d-glucopyranoside, (E)-dihydropinosylvin-3-O-*β*-d-glucopyranoside, (E)-pinosylvin-3-O-*β*-d-glucopyranoside, dihydropinosylvin, and pinosylvin [10–12]. Leaves were claimed for antioxidant activities, antiviral activities, especially chikungunya, and reduction of oxidative stress [13–16]. Young leaves, fruits, pods, and unripe seeds are edible and contained crude protein, ash, crude fiber, carbohydrates, amines, amides, carboxylic acids, and aromatic compounds [17]. Leaves contained important flavonoids, namely, chrysin, baicalein, baicalein-7-O-glucoside, baicalein-7-O-diglucoside, chrysin-7-O-glucuronide, baicalein-7-O-glucuronide, and a chrysin-diglucoside [18]. Root and root bark were reported for important flavonoids like baicalein, chrysin, oroxylin A, baicalein-6-O-glucoside, *β*-sitosterol, and its 3-O-glucoside, and stigmasterol-3-O-glucoside [19].

For bark or stem bark, many biochemical activities have been assessed like antimicrobial, antidiarrheal, analgesic, cytotoxic, hepatoprotective, gastroprotective, antiproliferative, antimetastatic, antiobesity potential, and antioxidant activities [20–31] and seed has antidiabetic potentials and showed synergistic potentials with acarbose [32]. Various flavonoids, namely, 5,7-dihydroxyflavone, 5,7-dihydroxy-3-methoxyflavone, 3,5,7-trihydroxyflavone, 5,7,4′-trihydroxy-3-methoxyflavone, 3,5,7,4′-tetrahydroxy flavone, and 5,7,4-trihydroxyflavone, were identified and separated from the stem bark portion of* O. indicum* and structure of isolated chemicals was characterized [33–40]. A number of flavonoids compounds had been separated and identified especially from bark (both stem and root) leaves and seeds. Most prominent flavonoids are baicalein, chrysin, ellagic acid, oroxylin A (baicalein-7-o-glucoside), oroxin A, 5-hydroxy-4, 7-dimethoxy flavone, 7-methoxy chrysin, etc. [41–47]. The whole stem of this miracle plant was claimed scientifically for regulation and expression of tumor necrosis factor alpha (TNF*α*), interleukin 6 (IL6), NFkB, and mitogen-activated protein kinase p38 (p38MAPK), associated with oxidative status in antitubercular therapy-induced hepatotoxicity [48].

Various research works had been conducted for the screening of toxicological studies of the whole part of* O. indicum* [49–53] and it was observed that dose of this plant (both aqueous and organic one) extract was used in a range from 10 mg/kg b.w. (p.o.) to 5000 mg/kg b.w. (p.o.) considered safe [16, 24, 54, 55]. But it was found that nitrosated ethanolic extract of the bark of* O. indicum* at a dose of 2000 mg/kg b.w. was genotoxic and mutagenic and toxicity increases on a dose-dependent manner up to 11 folds [52]. Experimental procedure confirmed that* O. indicum* stem bark extracts showed nonmutagenic, noncytotoxic, and nongenotoxic effects and toxicity information is not reported in any of scientific experiments after studying and comparing its safety profile with that of other plants [56].

After an extensive literature search, this study was aimed at investigating anti-inflammatory, antiulcer, antidiabetic, antihyperlipidemic, and liver enzyme (SGPT and SGOT) activities of stem bark of* O. indicum.*

## 2. Materials and Methods

### 2.1. Plant Collection and Processing

For the current investigation, the fresh and raw stem barks of* O. indicum* were collected during the months of January-February 2017 and authentication was identified by the National Herbarium with accession number DACB-43471. The stem barks were chopped followed by washing and drying at ambient temperature for 72 hours. To obtain powder drying, grinding and sieving of the dried portion were followed by weighing using an analytical balance.

### 2.2. Preparation of Crude Methanolic Extract

To prepare crude extract 150 g powdered material was soaked in methanol and the amount of methanol was 900 ml. The round amber color bottle was tightly sealed, kept for a total of 20 days accompanied by occasional shaking and stirring. By using Whatman filter paper filtration was carried. By using rotary evaporator at 7-8 rpm and with a temperature of 68°C crude methanolic extract of* O. indicum* (MEOI) was collected after evaporation of methanol. This generated extract was used for anti-inflammatory, antidiabetic, antihyperlipidemic, and liver enzyme (SGPT and SGOT) activities.

### 2.3. Preparation of Crude Ethanolic Extract and Its Fractions for Antiulcer Activities

For the preparation of crude extract dried stem bark was weighed for 150 g and grinded to 60 mesh size. Then the grinded materials were dissolved in 2 L ethanol (50% ethanol in water) for twenty days in a conical flask accompanied by occasional shaking and stirring. This was followed by filtration and the filtrate was dried using rotary evaporator (yield 18.34%). The extracted material was then extracted by petroleum ether (PET) followed by drying (yield 0.35% w/w). The residual material was then air-dried and liquid ammonia solution was used to moisten the dried material. The residual material again was extracted with chloroform (CLF) followed by drying (yield 1.90% w/w). Again the remaining unextracted material was then extracted with n-butanol (n-BuOH) and ethyl acetate (EtOAc) sequentially to obtain n-BuOH (yield 2.2% w/w) and EtOAc (yield 2.9% w/w) fractions. The dried fractions were conserved in air tight humidity protected borosilicate glass container.

### 2.4. Collection of Test Animals

A number of 400 test animals were purchased from the Research Institute International Centre for Diarrhoeal Disease Research, Bangladesh (ICDDR,B). Both Swiss Albino and Long Evans rats aged 4-6 weeks, having the weight of 200 to 220 g, were subjected for anti-inflammatory, antiulcerative, antidiabetic, and antidyslipidemic test. Liver enzyme concentration had also been tested. Groupings were done according to need for each test and protocols among with time duration. Rats were kept at ambient temperature (25-28°C) and maintained at dark/light cycles followed by acclimation of necessary duration. The standard pellet was purchased from ICDDR,B for their diet and additional fat-enriched diet supplied. Both sex rats were used but kept separately for the purpose of avoiding breeding. Animal ethics had been strictly maintained and handled them in the most human way. Coprophagy carefully was prevented. The animals groupings were done randomly. All reagents were prepared instantly. Within the whole study period, it was ensured that rats were healthy and playful. The cervical dislocation procedure was used to sacrifice all rats. Animals after sacrifice dumped according to rule [57–60]. Animal ethical committee approved the whole protocol.

### 2.5. Drugs and Chemicals for Anti-Inflammatory Activity

All solvents and reagents including *γ*-carrageenan were procured from Sigma-Aldrich, USA. Ibuprofen was a generous gift of Eskayef Pharmaceutical Company Ltd., Bangladesh.

### 2.6. Grouping of Animals

Swiss Albino rats aged 4-6 weeks having the weight of 180 to 200 g were subjected for anti-inflammatory study. Each group contained 6 rats. The animals were kept whole day fasting and only allowed drinking water before the experiment started and coprophagy was carefully prevented.

### 2.7. Anti-Inflammatory Activity

To measure anti-inflammatory activity the same procedure followed MEOI suggested by Winter* et al.* [61], by using carrageenan as an edematogenic agent after some modification. Six rats were in each group and a total of six groups were made. Group I was considered as control, treated with only 0.9% saline with 2-3 drops of tween 80. Group II was given 0.9% saline and 0.1 ml 1% carrageenan. Group III was treated with ibuprofen (10 mg/kg b.w.) as a reference drug. Group IV, group V, and group VI were treated with crude extract and doses were 100, 200, and 400 mg/kg b.w. individually. After one hour, each rat was injected 1% carrageenan dissolved in a saline solution into the right hind paw. The paw edema volume was computed every hour using slide calipers and the left hind paw was considered as a reference of the noninflamed paw for comparison. The following formula was used for calculation of percentage inhibition of paw edema:

(1) $$\begin{array}{c} Paw\;\;edema\;\;inhibition\;\;\left(\;\%\;\right)=\left[\;\frac{\left(V_{c}-V_{t}\right)}{V_{c}}\;\right]\times 100 \end{array}$$

Vc denotes the average paw volume of control animal and Vt represents the average paw volume of treated animal.

### 2.8. Histological Evaluation

To evaluate histopathological examination, rat paws were collected 6 hours after the induction with an inflammatory agent (carrageenan). The tissue slices (5 *μ*m thick) were processed for histological evaluation of inflamed paw 10% by keeping it within formalin buffer at 4°C. After that histopathology examination was done from the Histopathology Unit of Popular Hospital, Dhaka, Bangladesh.

### 2.9. Drugs and Chemicals for Antiulcer Activity

Omeprazole was collected from the Eskayef Pharmaceutical Company Ltd., Bangladesh. Solvents and reagents were confirmed to be of analytical grade. Oral administration of omeprazole was done by dissolving it in dimethyl sulfoxide (DMSO).

### 2.10. Grouping of Animals

Long Evans rats aged 4-6 weeks having the weight of 200 to 220 g were subjected for antiulcerative study. Each group contained 6 rats. The animals were kept fasting 24 hours before the experiment started and coprophagy was carefully prevented.

### 2.11. Antiulcer Activity

Pretreatment procedure was followed for examining mucosal damage in gastric cell lining. To do so, standard drug omeprazole and all distinguished doses of aforesaid test samples of* O. indicum* were administered. Ulcer model was induced by ethanol acid in a fixed dose measured in each rat except for normal control. The dose was 25 ml/kg of 0.3M hydrochloric acid in 60% ethanol [62]. Waiting till 90 minutes after persuasion of ulcer all rats were sacrificed and stomachs were collected and instantly excised along the larger curvature and washed. The magnifying lens was used to examine ulcers in gastric mucosa and scoring of ulcer calculated [63]. Crude EtOH of stem bark of* O. indicum* and its different fractions, namely, PET, CLF, EtOAc, and nBUT, was observed against damage of gastric mucosa. The methanol extract and its different fractions were used for finding out the ulcerative profile. Ulcer index was calculated by using the mean ulcer score. The ulcer protection percentage was estimated by using the following equation:

(2) $$\begin{array}{c} Ulcer\;\;protection\;\;\left(\;\%\;\right)=\frac{\left(U_{c}-U_{t}\times 100\right)}{U_{c}} \end{array}$$

U_c_ denotes the average ulcer index of control animal and U_t_ represents the average ulcer index of treated animal.

Lesions were assessed by unbiased neutral observers using a 7 binocular magnifier. Grouping of rats for antiulcerative activities was done as normal control, ethanol control, 100, 200, and 400 mg/kg b.w. per oral along with a dose of ulcer induction by ethanol acid.

### 2.12. Drugs and Chemicals for Antidiabetic, Antihyperlipidemic, and Liver Enzyme Activities

The drug glibenclamide and simvastatin both were obtained from Eskayef Pharmaceutical Company Ltd., Bangladesh. Total cholesterol TC, TG, LDL, HDL, SGPT, and SGOT kits and Alloxan were brought from Sigma-Aldrich, USA.

### 2.13. Grouping of Animals

A total of 84 Long Evans male rats were grouped for both glucose tolerance test and antidiabetic tests. In glucose tolerance test a total of 24 rats were used in four different groups after time intervals 0, 0.5, 1, and 2 hours, respectively. Other 60 rats were divided into five individual groups. Group I was considered as normal control. Group II was diabetic control. Group III was treated with standard glibenclamide. Group IV was treated with 400 mg/kg b.w. p.o. MEOI. Group V was treated with a combination of glibenclamide and 400 mg/kg b.w. p.o. of MEOI. The same grouping was repeated and implemented in case of finding hypolipidemic activities by simvastatin and experimental crude samples.

### 2.14. Preparation of Dosage of Active Drugs and Test Sample

Glibenclamide dissolved in DMSO and administered at a dose of 1.2 mg/70kg b.w. Crude of 100, 200, and 400 mg/kg b.w. p.o. doses was used and extracted by methanol from experimental plant* O. indicum*. For combination therapy the 0.6mg/70kg b.w. dose of glibenclamide and 400 mg/kg b.w. p.o. dose of* O. indicum* were used. Simvastatin dissolved in ethanol and ingested at a dose of 10 mg/70kg b.w. and for combination therapy the 5mg/70kg b.w. dose of simvastatin and 400 mg/kg b.w. p.o. dose of* O. indicum* were used.

### 2.15. Collection of Blood Serum and Liver

After finishing 8-week and 12-week treatment the rats were anesthetized with phenobarbital sodium. With heparinized syringe, an average of 4 ml blood was collected by cutting the thoracic artery and the collected sample was centrifuged at 3000 rpm for 20 minutes and serum was collected for the test.

### 2.16. Oral Glucose Tolerance Test

The oral glucose tolerance test (OGTT) was performed by the Vigneaud* et al*.'s [53] method. After overnight fasting, 1.5 gm/kg per oral glucose was administered and glucose level estimated in blood at 0, 0.5, 1, and 2 hours' interval. Glibenclamide, a blood glucose lowering drug, was administered (at a dose of 1.2mg/70kg b.w. per oral) two hours prior to glucose intake.

### 2.17. Antihyperglycemic and Antihyperlipidemic Activities

Five different groups were prepared for testing the antihyperglycemic effect. A total of 12 hours of starvation were maintained for experimental rats. All the rats were tested for a baseline glucose level using a glucometer. The first group for the normal group received no drug, the second group was selected for the diabetic control group, and the third group stands for glibenclamide received a dose of 1.2 mg/70kg b.w. The fourth group stands for methanolic extract of* O. indicum* at a dose of 400 mg/kg b.w., p.o. The fifth group received the combination of drugs (glibenclamide and MEOI) mentioned earlier.

For hypolipidemic activities, the same grouping and procedure were followed with a dose of 10 mg/70kg b.w. and combination therapy received the 5mg/70kg b.w., the dose of simvastatin, and 400 mg/kg b.w., p.o., dose of* O. indicum*.

#### 2.17.1. Eight- and Twelve-Week Repeated Dose Treatment of Glibenclamide, MEOI, and Combination of Both

For the determination of blood glucose level in the diabetic rat model induced by alloxan, 8- and 12-week treatment protocols were conducted. According to this procedure glibenclamide, MEOI, and combination of both (glibenclamide and MEOI) were administered daily and after completion of treatment glucose level was detected by glucometer.

#### 2.17.2. Eight- and Twelve-Week Repeated Dose Treatment of Simvastatin for Hyperlipidemia, SGPT, and SGOT Levels

Collected serum from thoracic artery was subjected to various lipid levels. Level of TC, TG, LDL, and HDL in serum and level of SGPT and SGOT were also determined by diagnostic kit manufactured by Sigma-Aldrich, USA. For performing these tests the International Federation of Clinical Chemistry (IFCC) standard was followed [64].

### 2.18. Statistical Analysis

To represent glucose level and lipid and liver enzyme level all comparable results were expressed by mean±SEM. For this purpose GraphPad Prism software was used. Data were analyzed by ANOVA followed by Dunnett's multiple comparison test, to find out the significance level of *p* values.

## 3. Results

### 3.1. Evaluation of Anti-Inflammatory Activity

After treatment, it was observed that reference standard drug ibuprofen (20 mg/kg b.w., p.o.) and all different doses of methanolic extract (100, 200, and 400 mg/kg b.w., p.o.) revealed considerable depletion in paw thickness in a total duration period of six hours (Table 1). Compared to carrageenan control it was seen that all different doses of extract showed considerable anti-inflammatory activity which was significant (*p*< 0.01). After completion of study in 6 hours it was noticed that ibuprofen (20 mg/kg b.w.) at the end of the study (20 mg/kg b.w.) spotted 53.33% of inhibition and all methanolic extracts (100, 200, and 400 mg/kg b.w.) decreased edema in mice paw by 37.50%, 48.34%, and 55.83% individually.

In naked eyes, it was observed that, after induction of carrageenan, edema formation was noticeably observable characterized by redness and swelling in all six groups, namely, normal control (NC), carrageenan control (CC), ibuprofen 20 mg/kg b.w. (IP), 100 mg/kg b.w./day methanolic extract of* O. indicum* (MEOI-100), 200 mg/kg b.w./day methanolic extract of* O. indicum* (MEOI-200), and 400 mg/kg b.w./day methanolic extract of* O. indicum* (MEOI-400) groups. In each case, inflammatory responses were characterized by swelling, redness, and edema formation symptoms.

### 3.2. Histopathology of Tissue Obtained from Paw

**Figure 1:**
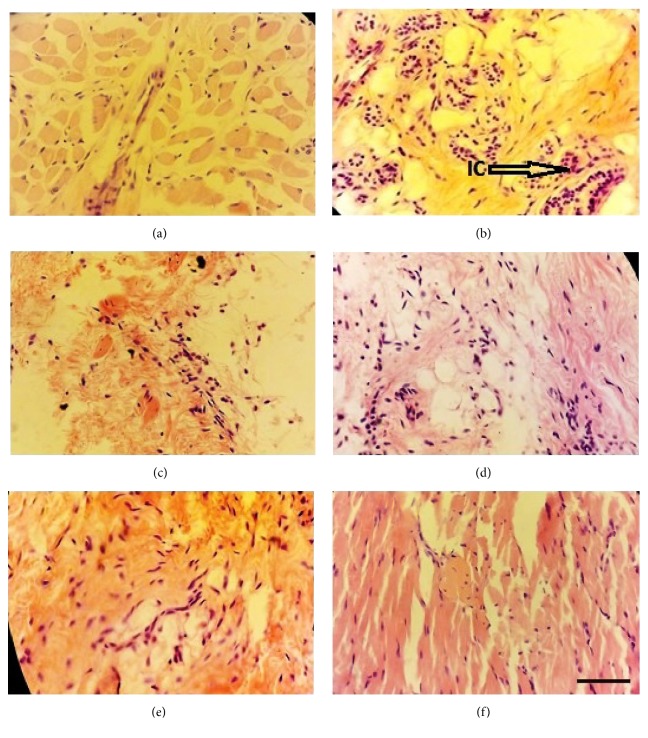
*Histological analysis of paw tissue in the carrageenan-induced paw edema model*. Each picture is representative of each group as follows: (a) normal control, (b) carrageenan control, (c) ibuprofen 20 mg/kg b.w., (d) 100 mg/kg b.w./day methanolic extract of* O. indicum*, (e) 200 mg/kg b.w./day methanolic extract of* O. indicum*, and (f) 400 mg/kg b.w./day methanolic extract of* O. indicum*. IC, inflammatory cells; arrow indicates infiltration of inflammatory cells. Each group was assessed at 400X magnification, and the scale bar is 40*μ*m.

After histopathological examination (Figure 1) it was observed that as reference standard drug ibuprofen, the highest dose extract of* O. indicum* (400 mg/kg b.w.), put down the cell infiltration and suppressed inflammation. But more tissue level analysis needs to explain with extract mechanism how the extract of MIOE works. The photomicrograph from paw tissue of normal control group rat showed only minimal inflammatory cells infiltration (Figure 1(a)) whereas the photomicrograph from paw tissue of carrageenan control group showed numerous infiltrations of inflammatory cells (arrow) (Figure 1(b)). The photomicrograph from paw tissue of the ibuprofen treated group showed minimal inflammatory cells (Figure 1(c)). On the other hand, the photomicrograph from methanolic extract treated groups (100 and 200 mg/kg b.w.) showed a moderate number of inflammatory cells (Figures 1(d) and 1(e), resp.). However, the photomicrograph from the highest dose extract of* O. indicum* (400 mg/kg b.w.) put down the cell infiltration and suppressed inflammation (Figure 1(f)).

### 3.3. Evaluation of Antiulcer Activity

For the study of gastric mucosal damage crude extract in ethanol and different fractions, namely, PET, CLF, EtOAc, and nBUT, was monitored. One hour after inducing an ulcer, animals were sacrificed. The stomachs were excised and were opened over the greater curvature and rinsed with saline solution (0.9%) to remove the blood clots. Thereafter, each gastric sample was placed on a slide. The gastric damage area was firstly observed by Tripod magnifier (10 × magnification) for primary observation before being subjected for histopathological studies. Close monitoring revealed that ulcer formation in the gastric layer was potentiated by acute to subchronic gastritis. This was characterized by hemorrhagic lesions followed by damage of mucosal capillaries, increased permeability, and aggregation of platelets and clot formation. Stomachs obtained from the normal control group were healthy and viable and no clot formation occurred under cell line. But in ethanol control group stomachs were perforated, red marked, and were frequently identified due to hemorrhagic lesions and capillary rupture. For treatment with doses of 100 and 200 mg/kg b.w./day both group stomachs were examined with gastric lesion and mildly notable blood clot formation due to the injury of cell line layer. But in the stomachs treated with 400 mg/kg b.w./day dose of* O. idcicum* there were no noticeable perforation, blood clot formation, and cell damage. Stomachs were healthy and in fine fettle compared to the control group.

It was showed (Table 2) that control group indicated ulcer index 7.04±0.112 with zero percentage of inhibition. But nBUT and PET showed 0.07±0.007 and 0.27±0.011 ulcer index with inhibitory percentage of 99 and 96, respectively. Among all EtOAc showed a lower percentage of inhibition 86 with ulcer index 0.87±0.044. Standard drug omeprazole gave 88.70% inhibition effect against ulcer.

### 3.4. Evaluation of Blood Glucose Level

Table 3 manifested the fasting plasma glucose level in glucose-induced hyperglycemia. It was observed that blood glucose level decreased from 10.57 ± 0.32 to 7.55 ± 0.22 mmol/L after 30 minutes in comparison with the control group.

### 3.5. Evaluation of Antihyperglycemic, Antihyperlipidemic, and Liver Enzyme Activities

#### 3.5.1. Effects on Glucose Level in Rats in 8 and 12 Weeks of Treatment by Glibenclamide MEOI and Combination Therapy

Glibenclamide alone revealed (*p<0.05*) reduction in glucose level to 7.31 ± 0.21 from 16.28 ± 0.12 mmol/L after 8 weeks of treatment (Table 4). But 400 mg/kg b.w., p.o. dose of MEOI suppressed glucose level in blood only by 26% but not remarkably. Combination therapy, on the other hand, reduced blood glucose level to a normal extent by 61%. Glibenclamide alone indicated notable (*p<0.05*) downturn in glucose level from 33.5 ± 0.31 to 7.9 ± 0.19 mmol/L after completing 12 weeks of treatment in diabetic rats (Table 5). At the dose of 400 mg/kg b.w., p.o. of MEOI there was decrease in blood glucose level by 37% whereas glibenclamide was lessened to 77% in blood glucose level which was noteworthy. Combination therapy, on the other hand, lowered blood glucose level to a normal extent by 79%. Combination dose (glibenclamide+ 400 mg/kg b.w. of MEOI) showed nearly the same result as glibenclamide did and did not exhibit any additive or synergistic effect in both 12- and 8-week treatment protocols.

#### 3.5.2. Effects on Lipid Profile in Rats in 8- and 12-Week Treatment by Simvastatin, MEOI, and Combination Therapy

Tables 6, 7, 8, and 9 demonstrated lipid-lowering ability of simvastatin, MEOI, and combination of both drugs after repeated dose treatment for 8 and 12 weeks on the lipid profile in diabetic rats model induced by alloxan. After 8 weeks of treatment, it was revealed that simvastatin, MEOI, and combination of both drugs reduced total cholesterol level by 40%, 22%, and 44% (Table 6), respectively, but in 12-week treatment total cholesterol level was reduced by 44%, 28%, and 48% after applying the same dose of simvastatin, MEOI, and combination of both drugs. In 8- and 12-week treatment protocol triglyceride levels were reduced by 32%, 15%, and 35% and 32%, 17%, and 36%, respectively (Table 7). LDL-cholesterol levels were reduced by 28%, 4%, and 31% in 8-week treatment protocol while in 12-week treatment protocol levels were reduced by 28%, 5%, and 33% (Table 8). In 8-week treatment protocol both HDL levels were increased by 34%, 13%, and 36% and in 12 weeks increased by 36%, 8%, and 38% consequently (Table 9).

#### 3.5.3. Effect on Elevated Liver Enzyme Levels

Tables 10 and 11 illustrated the effects of glibenclamide, 400 mg/kg b.w., p.o. of MEOI and combination dose on elevated liver enzyme level in diabetic rats. After 8-week treatment with the glibenclamide, 400 mg/kg b.w., p.o. of MEOI and combination (glibenclamide, 400 mg/kg b.w., p.o. of MEOI) of both drugs it was seen that concentration of SGPT (ALAT) showed downturn by 41.98%, 7.27%, and 54.61% and SGOT (ASAT) by 41.35%, 4.74%, and 42.30% in comparison with control group individually (Table 10).

After 12-week treatment with the glibenclamide, 400 mg/kg b.w., p.o. of MEOI and combination (glibenclamide, 400 mg/kg b.w., p.o. of MEOI) of both drugs it was exhibited that concentration of SGPT (ALAT) showed downturn by 43.23%, 8.01%, and 54.86% and SGOT (ASAT) by 42.40%, 5.31%, and 44.85%, respectively (Table 11).

## 4. Discussion

Phytoconstituents are the backbone for exerting copious therapeutic actions and aid as a blueprint for the discovery and development of new pharmaceuticals [65–67]. In this study, anti-inflammatory, antiulcer, antidiabetic, antihyperlipidemic, and liver enzyme activities of stem bark of* O. indicum* were investigated.

In case of anti-inflammatory activities, the edema in the right paws could be segmented into the first phase followed by the second phase [68, 69]. In phase one, edema formation occurred instantaneously after carrageenan injection. The first phase counted for two hours approximately. Phase two counted for two to up to six hours and larger edema formation occur characterized by redness and swelling. This inflammatory action was due to various inflammatory chemicals like prostaglandin, prostacyclin, and leukotriene followed by the pathway COX 2 mechanism [22, 70–73]. Noticeably Figure 1(a) group was distilled water treated paw with no carrageenan injection which demonstrated damaged tissue and was conserved for the identification of dermal collagen and leukocytes. Figure 1(b) indicated swelling and redness after carrageenan injection induction due to diapedesis and chemotaxis by inflammatory cells including white blood cells and red blood cells. Figure 1(c) denoted treatment with ibuprofen (20 mg/kg b.w.) with slight inflammatory cells appearance. Figures 1(d) and 1(e) both indicated that methanolic extract with doses of 100 and 200 mg/kg b.w. showed mild to moderate cell infiltration. Followed by treatment with the dose of 400 mg/kg b.w. in Figure 1(f) it was observed that cells' infiltration was nearly abolished. The extract of MEOI showed a considerable anti-inflammatory effect. Also, the histopathological examination of paws revealed that methanolic extract of* O. indicum* reduced aggregation of inflammatory cells. All studies support previous experiment with different parts (seed) of the same plant. All these observations in different groups showed similarities with previous studies [74–83].

In case of screening of antiulcer property, ethanol was used to produce mucosal damage in the stomach layer. Extract of* O. indicum* (with the dose 400 mg/kg b.w., p.o.) exhibited a substantial downturn for reduction of gastric ulceration. Omeprazole was used as a standard drug and after treatment procedure it was observed that standard omeprazole, PET, and nBUT showed a considerable reduction in ulceration. The extract was used at a dose of 400 mg/kg b.w. and among all fractions the PET (96%) and nBUT (99%) fractions manifested sharp reticence of gastric lesions against mucosal damage induced by ethanol acid. The comparison was done with reference standard drug omeprazole and the ulcer model after treatment with both active fractions and omeprazole showed dramatic gastric mucosal damage conservation. This study also supports previously done experimental studies [46, 84–93].

In case of antidiabetic activity, glibenclamide is considered as good glucose level lowering drug and exhibited significance. In both treatment procedures, namely, 8 weeks and 12 weeks, individual dose of extract of* O. indicum* revealed reduction of blood glucose level but it was not noteworthy on the contrary that combination dose (for both 8 and 12 weeks) was able to decrease glucose level in a significant extent. A number of flavonoids compounds had been separated and identified especially from bark (both stem and root) leaves and seeds. Two chemicals, namely, oroxylin A (baicalein-7-o-glucoside) and oroxin A, have antidiabetic potentials [46, 47]. These phenomena support the previous experiment as among all constituents oroxylin A and oroxin A both have insulin resistance potentials and have capability of inhibiting *α* glucosidase [32, 46, 47]. It was seen that extracted constituent revealed more activity than the crude extract. In both time- and dose-dependent treatment protocols it was monitored that long-term (12 weeks) treatment showed a drop of glucose level compared to short-term (8 weeks) treatment. In both cases only combination therapy showed significance and was supported by preceding studies [46, 47, 94–101].

For the assessment of hypolipidemic activity, the extract alone (400mg/kg b.w., p.o.) failed to decline TC, TG, and LDL level by remarkable level. But a mild drop of lipid level occurred in each case. Only in case of 12-week treatment combination therapy showed significance in lowering lipid level. In case of HDL level elevation combination therapy indicated a considerable upward trend. This study also supports the previous experiment as simvastatin was treated as a very potential lipid-lowering drug and coincided with prior studies [102–106]. To identify the level enzymes (both SGPT and SGOT) both treatment protocols failed to decrease the level of specific enzyme level to a noteworthy extent and exhibited similarities with past studies [107–113] but revealed satisfactory decline. Liver enzymes SGPT and SGOT are considered as surrogate markers of diabetes mellitus complication. This study also showed that stem bark alone has no significant effect on liver enzyme concentration. For liver enzyme concentration assessment, neither individual dose of MEOI nor combination did show any significant reduction. Cases of TC, TG, LDL, and HDL level except simvastatin which was used as a standard drug and combination of simvastatin and 400 mg/kg b.w. MEOI showed significance but the dose of extract at 400mg/kg b.w alone did not show any significance.

## 5. Conclusion

Extract of* O. indicum* exhibited potent and remarkable anti-inflammatory and antiulcer activities. On the other hand, it showed moderate antidiabetic and antidyslipidemic properties and liver enzymes' concentration lowering effects. However, the mechanisms behind all those effects are still not in daylight. Therefore, further experiments should be carried out for the purpose of the phytoconstituents and expected mechanisms that are subjected and interrelated to the constituents to exhibit various effects in both* in vivo* and* in vitro* experimental models.

## Figures and Tables

**Table 1:** Anti-inflammatory effect of MEOI on carrageenan-induced changes in paw thickness in experimental rats.

Groups	NC	CC	IP-20	MEOI-100	MEOI-200	MEOI-400
Dose (mg/kg b.w.)	–	–	Ibuprofen 20	Extract 100	Extract 200	Extract 400
Initial paw thickness	0.54±0.04	0.43±0.07	0.51±0.06^*∗*^	0.52±0.04^*∗*^	0.47±0.07^*∗*^	0.50±0.04^*∗*^
Paw thickness after 1 hr	0.54±0.03	0.69±0.05^*∗*^	0.60±0.04^*∗*^	0.68±0.07	0.64±0.05	0.59±0.06^*∗*^
Paw thickness after 2 hrs	0.54±0.04	0.85±0.05^*∗∗*^	0.64±0.05^*∗∗*^	0.75±0.04	0.68±0.05^*∗*^	0.62±0.06^*∗∗*^
Paw thickness after 3 hrs	0.54±0.05	0.98±0.06^*∗∗*^	0.68±0.07^*∗∗*^	0.80±0.05	0.72±0.06^*∗*^	0.65±0.05^*∗∗*^
Paw thickness after 4 hrs	0.54±0.06	1.15±0.08^*∗∗*^	0.70±0.06^*∗∗*^	0.83±0.07^*∗*^	0.75±0.07^*∗∗*^	0.67±0.09^*∗∗*^
Paw thickness after 5 hrs	0.54±0.05	1.18±0.09^*∗∗*^	0.63±0.07^*∗∗*^	0.79±0.09^*∗*^	0.71±0.06^*∗∗*^	0.61±0.06^*∗∗*^
Paw thickness after 6 hrs	0.54±0.06	1.20±0.07^*∗∗*^	0.56±0.06^*∗∗*^	0.75±0.08^*∗∗*^	0.62±0.07^*∗∗*^	0.53±0.07^*∗∗*^

Observed values were represented by mean ± SEM (*n=*6). ^*∗*^*p* < 0.05 and ^*∗∗*^*p* < 0.01 when compared with control group.

NN = normal control, CC = carrageenan control, IP = ibuprofen 20 mg/kg b.w., MEOI-100 = 100 mg/kg b.w./day methanolic extract of *O. indicum*, MEOI-200=200 mg/kg b.w./day methanolic extract of *O. indicum*, and MEOI-400 = 400 mg/kg b.w./day methanolic extract of *O. indicum*.

**Table 2:** Antiulcer effect of EtOHand its fractionsin experimental rats.

Groups	Ulcer indexed	Ulcer inhibition (%)
Control	7.04±0.112	–
EtOH (50%)	0.65±0.035	89.29
EtOAc	0.87±0.044	86.12
CLF	0.58± 0.05	91.50
PET	0.27±0.011	95.80
nBUT	0.07±0.007	98.60
Omeprazole	0.75±0.042	88.70

Observed values were represented by mean ± SEM (n=6).

**Table 3:** Effect of glibenclamide on blood glucose level.

Time (hour)	Glucose control (mmol/L)	Glucose+Glibenclamide (mmol/L)
0	6.23±0.17	6.78±0.27
0.5	10.57±0.32	7.55±0.22
1	7.66±0.17	6.1±0.19
2	6.12±0.08	5.34±0.03

Observed values were represented by mean ± SEM (n=6).

**Table 4:** Effect of glibenclamide, MEOI, and combination therapy on blood glucose level in 8-week treatment protocol.

Time duration	*Blood glucose level (mmol/L)*				
	Control	Diabetic control	Alloxan+Glibenclamide	Alloxan+400 mg/kgb.w.	Alloxan+Combination
Before 8 weeks	6.58 ± 0.09	16.28 ± 0.12	16.36 ± 0.14	16.41 ± 0.15	16.2 ± 0.13
After 8 weeks	6.53 ± 0.23	16.75 ± 0.23	7.31 ± 0.21	12.2 ± 0.17	6.4 ± 0.09^*∗*^

Observed values were represented by mean ± SEM (n=6). ^*∗*^*p* < 0.05 when compared with control group.

**Table 5:** Effect of glibenclamide, MEOI, and combination therapy on blood glucose level in 12-week treatment protocol.

Time duration	*Blood glucose level (mmol/L)*				
	Control	Diabetic control	Alloxan+Glibenclamide	Alloxan+400 mg/kg b.w.	Alloxan+Combination
Before 12 weeks	6.43 ± 0.29	34.58 ± 0.12	33.50 ± 0.31	32.96 ± 0.34	34.50 ± 0.21
After 12 weeks	6.58 ± 0.23	34.73 ± 0.45	7.90 ± 0.19	20.34 ± 0.13	7.30 ± 0.10^*∗*^

Observed values were represented by mean ± SEM (n=6). ^*∗*^*p* < 0.05 when compared with control group.

**Table 6:** Effect of simvastatin, MEOI, and combination therapy on total cholesterol level in 8- and 12-week treatment protocol.

Time duration	*TC (mg/dl)*				
	Control	Diabetic control	Alloxan+Simvastatin	Alloxan+400 mg/kg b.w.	Alloxan+Combination
TC in 8 weeks	165.25 ± 2.2	263 ± 3.45	158 ± 2.39	206 ± 2.1	148 ± 2.0
TC in 12 weeks	165 ± 1.90	278 ± 3.10	156 ± 1.97	201 ± 1.1	144 ± 0.90^*∗*^

Observed values were represented by mean ± SEM (n=6). ^*∗*^*p* < 0.05 when compared with control group.

**Table 7:** Effect of simvastatin, MEOI, and combination therapy on triglycerides level in 8- and 12-week treatment protocol.

Time duration	*TG (mg/dl)*				
	Control	Diabetic control	Alloxan+Simvastatin	Alloxan+400 mg/kg b.w.	Alloxan+Combination
TG in 8 weeks	120.5 ± 8.08	193.5 ± 2.39	131.4±2.01	164.5 ± 1.31	125 ± 1.1
TG in 12 weeks	125 ± 0.23	192.2 ± 0.23	130± 0.21	160 ± 0.13	123 ± 0.12^*∗*^

Observed values were represented by mean ± SEM (n=6). ^*∗*^*p* < 0.05 when compared with control group.

**Table 8:** Effect of simvastatin, MEOI, and combination therapy on low-density lipoprotein level in 8- and 12-week treatment protocol.

Time duration	*LDL (mg/dl)*				
	Control	Diabetic control	Alloxan+Simvastatin	Alloxan+400 mg/kg b.w	Alloxan+Combination
LDL in 8 weeks	124 ± 2.4	185.75 ± 2.7	135 ± 1.4	178 ± 1.4	128 ± 1.2
LDL in 12 weeks	125.75±2.1	182.25 ± 2.3	130.5±1.2^*∗*^	173 ± 1.1	123 ± 1.1^*∗*^

Observed values were represented by mean ± SEM (n=6). ^*∗*^*p* < 0.05 when compared with control group.

**Table 9:** Effect of simvastatin, MEOI, and combination therapy on high-density lipoprotein level in 8- and 12-week treatment protocol.

Time duration	*HDL (mg/dl)*				
	Control	Diabetic control	Alloxan+Simvastatin	Alloxan+400 mg/kg b.w.	Alloxan+Combination
HDL in 8 weeks	55.25±1.25	34.75 ± 0.85	52.5 ± 0.83	40.25 ±0.64	55 ± 0.5
HDL in 12 weeks	54.41±1.10	36.50 ± 1.19	42 ± 0.47	55.59 ± 0.85	58 ± 0.45^*∗*^

Observed values were represented by mean ± SEM (n=6). ^*∗*^*p* < 0.05 when compared with control group.

**Table 10:** Effect on liver enzyme concentration fluctuated after 8 weeks of treatment.

Liver enzyme concentration (U/L)	*Treatment group for 8 weeks*				
	Normal	Alloxan	Alloxan+Glibenclamide	Alloxan+MEOI 400mg/kg b.w.	Alloxan+Combination
SGPT	25.65 ± 0.63	77.11 ± 1.36	44.74 ± 0.95	71.5 ± 0.87	35.00 ± 0.98
SGOT	34.65 ± 0.51	73.75 ± 0.35	43.25 ± 0.58	70.25 ± 0.55	42.55 ± 0.76

Observed values were represented by mean ± SEM (n=6).

**Table 11:** Effect on liver enzyme concentration fluctuated after 12 weeks of treatment.

	*Treatment groups for 12 weeks*				
Liver enzyems concentration (U/L)	Normal	Alloxan	Alloxan+Glibenclamide	Alloxan+MEOI 400mg/kg b.w.	Alloxan+Combination
SGPT	24.75±0.63	73.11±1.26	41.50±0.85	67.25±0.64	33.00±1.47
SGOT	32.34±0.41	75.25±0.85	43.34±0.48	71.25±0.85	41.50±0.85

Observed values were represented by mean ± SEM (n=6).

## Data Availability

The experimental data obtained from various rat models used to support the findings of this study are available from the corresponding author upon request.

## Abbreviation

SGPT: Serum glutamic pyruvic transaminase
SGOT: Serum glutamic oxaloacetic transaminase
ALAT: Alanine aminotransferase
ASAT: Aspartate transaminase
OI: *Oroxylum indicum*
OGTT: Oral glucose tolerance test
MEOI: Methanolic extract of* Oroxylum indicum*
b.w.: Body weight
p.o.: Per oral
EtOH: Ethanol
PET: Petroleum ether
CLF: Chloroform
EtOAc: Ethyl acetate
nBUT: n-Butanol
mmol/L: Millimole/litre
TC: Total cholesterol
TG: Triglycerides
LDL: Low-density lipoprotein
HDL: High-density lipoprotein
ICDDR,B: International Centre for Diarrhoeal Disease Research, Bangladesh
IFCC: International Federation of Clinical Chemistry
DMSO: Dimethyl sulfoxide
NC: Normal control
CC: Carrageenan control
IP: Ibuprofen
SEM: Standard error mean
TNF*α*: Tumor necrosis factor alpha
IL6: Interleukin 6
p38MAPK: Mitogen-activated protein kinase p38.
